# Association between cerebrovasoreactivity and stroke in cerebral autosomal dominant arteriopathy with subcortical infarcts and leukoencephalopathy

**DOI:** 10.3389/fneur.2022.1087220

**Published:** 2023-01-09

**Authors:** Mao Mukai, Ai Hamano, Ikuko Mizuta, Isao Yokota, Akiko Watanabe-Hosomi, Hiraku Matsuura, Takashi Koizumi, Jun Matsuura, Tomoyuki Ohara, Shigenori Matsushima, Satoshi Teramukai, Kei Yamada, Toshiki Mizuno

**Affiliations:** ^1^Department of Neurology, Graduate School of Medical Science, Kyoto Prefectural University of Medicine, Kyoto, Japan; ^2^Department of Biostatistics, Graduate School of Medicine, Hokkaido University, Sapporo, Japan; ^3^Department of Anatomy and Neurobiology, Graduate School of Medical Science, Kyoto Prefectural University of Medicine, Kyoto, Japan; ^4^Department of Radiology, Graduate School of Medical Science, Kyoto Prefectural University of Medicine, Kyoto, Japan; ^5^Department of Biostatistics, Graduate School of Medical Science, Kyoto Prefectural University of Medicine, Kyoto, Japan

**Keywords:** cerebral autosomal dominant arteriopathy with subcortical infarcts and leukoencephalopathy, stroke, cerebrovasoreactivity, single-photon emission computed tomography, acetazolamide

## Abstract

**Background:**

Impaired cerebrovasoreactivity is thought to play an important role in the pathophysiology of cerebral autosomal dominant arteriopathy with subcortical infarcts and leukoencephalopathy (CADASIL). We aimed to clarify the association between cerebrovascular reactivity and stroke in patients with CADASIL.

**Methods:**

We retrospectively recruited 14 patients with CADASIL, eight of whom had symptomatic stroke. They underwent quantitative single-photon emission computed tomography using an autoradiographic method at rest and after acetazolamide (ACZ) administration. Regional cerebral blood flow (rCBF) in the cerebral cortex, lenticular nucleus, thalamus, and cerebellum was measured. We compared the rCBF parameters between patients with and without stroke.

**Results:**

The baseline characteristics and magnetic resonance imaging findings were similar between the two groups, except for a higher frequency of pyramidal tract sign (75% vs. 0%) and a larger number of old lacunes (15.4 ± 8.8 vs. 2.2 ± 1.8) in the patients with stroke. Of the rCBF parameters measured, significantly lower flow (mL/100 g/min) was observed in ACZ-rCBF in the thalamus (35.6 ± 9.4 vs. 51.1 ± 7.6, *p* = 0.01) and ΔrCBF in the thalamus (10.6 ± 3.7 vs. 21.0 ± 7.9, *p* = 0.02) in the patients with stroke.

**Conclusion:**

Cerebrovasoreactivity in the thalamus was significantly associated with stroke in patients with CADASIL.

## Introduction

Cerebral autosomal dominant arteriopathy with subcortical infarcts and leukoencephalopathy (CADASIL), caused by *NOTCH3* mutations, is one of the most common hereditary small vessel diseases (1). CADASIL is clinically characterized by migraine with aura, cerebral ischemic events, apathy, mood disturbance, and cognitive decline, progressing to dementia. Typical neuroimaging features include white matter lesions (WML) and subcortical infarcts (2). Cerebral ischemic events are the most frequent symptoms in patients with CADASIL, occurring in 60–85% of cases (2). Most patients experience recurrent stroke episodes, leading to irreversible dysfunctions such as gait disturbance, pseudobulbar palsy, and vascular dementia.

CADASIL arteriopathy is characterized by the loss of vascular smooth muscle cells and the thickening of the vascular wall, which are thought to impair vascular function (2, 3). Impaired cerebrovasoreactivity is thought to play an important role in the pathophysiology of CADASIL based on animal model studies (3) and comparisons between patients and controls (4, 5). Cerebrovasoreactivity is a hemodynamic biomarker that can be monitored using several instruments. Previous studies have examined cerebrovasoreactivity in patients with CADASIL using single-photon emission computed tomography (SPECT), positron emission tomography (PET), magnetic resonance imaging (MRI), or Doppler sonography (4, 6–11). Pfefferkorn et al. reported that cerebrovasoreactivity in response to CO_2_ was significantly reduced in patients with CADASIL compared to controls (7). Some studies have analyzed MRI parameters and cerebrovascular reactivity in relation to disease progression. Cerebral hypoperfusion and impaired cerebrovasoreactivity were associated with cognitive dysfunction in patients with CADASIL (5, 12). Liem et al. reported that lower cerebrovasoreactivity at baseline was associated with a larger increase in white matter hyperintensity (10). However, to our knowledge, cerebrovasoreactivity in relation to the occurrence of symptomatic stroke in patients has not yet been reported. To address this issue, we compared regional CBF (rCBF) at rest and after the administration of acetazolamide (ACZ) between CADASIL patients with and without stroke.

## Materials and methods

### Subjects

Fourteen patients with CADASIL who underwent ACZ challenge tests between January 2005 and December 2015 were recruited for this study. If a patient underwent multiple ACZ challenge tests, the last test was used in the analysis. Clinical information, including neurological findings, medical treatment, vascular risk factors, MRI findings, and ischemic events, was collected. Symptomatic stroke was defined as having both neurological signs and imaging findings indicative of stroke. Based on the records of symptomatic stroke before the SPECT study, the patients were divided into two groups: those with stroke (*n* = 8) and those without stroke (*n* = 6). Eight patients with stroke were further divided into two groups according to critical events, including symptomatic stroke during the 2 years after the SPECT study: those with critical events (*n* = 4, three patients with recurrence of symptomatic stroke and one patient deceased suddenly) and those without critical events (*n* = 4). The patients were suspected as CADASIL according to history of stroke, neurological symptoms, MRI findings, and family history. All the patients were definitively diagnosed with CADASIL based on the presence of typical cysteine-related mutations in the EGF-like repeat domain of NOTCH3.

### Measurements of regional cerebral blood flow (rCBF)

Measurements of rCBF after ACZ administration (ACZ-rCBF) and at rest (REST-rCBF) were performed using quantitative SPECT with the ARG method (13). Acetazolamide (1 g) was administered intravenously, starting 10 min before the intravenous injection of 185 MBq ^123^I-IMP (Nihon Medi-physics, Hyogo, Japan). After 10 min, one-point arterial blood sampling was performed from the brachial artery to assess whole-blood radioactivity concentration. The SPECT study was started 22 min after ^123^I-IMP injection and was conducted for 16 min using a triple-head gamma camera system (IRIX; Philips Healthcare, Cleveland, Ohio, USA) equipped with low-energy, parallel collimators. SPECT images with a 128 × 128 matrix were reconstructed using ordered-subset expectation maximization reconstruction with four iterations and 12 subsets. Attenuation correction was performed using Chang's method, and scatter was corrected using the triple-energy window method. To quantify rCBF, ^123^I-IMP SPECT images were analyzed using a three-dimensional stereotactic region of interest (ROI) template 3DSRT (FUJIFILM RI Pharma, Tokyo, Japan) (14).

3DSRT is composed of 318 ROIs in 12 segments (1: callosomarginal, 2: precentral, 3: central, 4: parietal, 5: angular, 6: temporal, 7: posterior, 8: pericallosal, 9: lenticular nucleus, 10: thalamus, 11: hippocampus, and 12: cerebellum) on each side. We measured the rCBF values (mL/100 g/min) of the 636 ROIs of patients with CADASIL and calculated the area-weighted average of the four main regions: cortex, lenticular nucleus, thalamus, and cerebellum. The cerebral cortex consists of the callosomarginal, precentral, central, parietal, angular, temporal, posterior, pericallosal, and hippocampal regions. REST-rCBF was measured 19.6 ± 12.9 (5–39) days before the ACZ-rCBF test.

The increase in rCBF (ΔrCBF) and regional cerebrovasoreactivity (rCVR) were calculated as follows:


$$\begin{array}{l} \text{ΔrCBF}\left(\text{mL/100 g/min}\right)\text{ = ACZ-rCBF − REST-rCBF }\\ \text{rCVR}\left(\%\right)\text{ = }(\left[\text{ACZ-rCBF − REST-rCBF}\right]\text{/REST}\text{ -rCBF})\;\;\\ \;\times 100 \end{array}$$

### MR imaging

MRI was performed using a 1.5T MR instrument (Gyroscan Intera Nova; Philips Medical Systems, Best, Netherlands). The severity of WML on fluid-attenuated inversion recovery images was classified into four grades according to a previous report (15). Specifically, T2-WI images were used to evaluate white matter lesions on four grades: A (no lesion), B (punctiform or slight periventricular hyperintensities, or both), C (nodular or moderate periventricular hyperintensities, or both) and D (confluent lesions or severe periventricular hyperintensities, or both) (15). White matter hyperintensity (WMH) and total brain parenchyma volumes were calculated using a 3D slicer (http://www.slicer.org). Old lacunes were identified as hypointense lesions on T1-weighted images with a signal identical to that of cerebrospinal fluid, sharp delineation, and a diameter >2 mm.

### Statistical analysis

The demographics, CBF parameters, and MRI parameters were compared between patients with and without symptomatic stroke, which were diagnosed based on neurological signs and MR findings. Fisher's exact test or Wilcoxon rank sum test was used. We further analyzed association between representative rCBF parameter and occurrence of critical events (symptomatic stroke or death) during two years after the SPECT examination in the patients with stroke. Univariate logistic regression models were used to calculate non-adjusted odds ratios with 95% confidence intervals (CIs) Receiver operating characteristic analysis/area under the curve (AUC) was used to assess relevance of the parameter. All the statistical methods were performed using JMP 14.2.0 (SAS Institute Japan Ltd., Japan). Statistical significance was set at *P* < 0.05.

## Results

### Baseline characteristics and MRI findings of the patients

Fourteen patients (nine males and five females; mean age: 56.3 ± 10.2 years) were included in this study. In the patients with symptomatic stroke, the intervals between past symptomatic stroke to SPECT examination were 1.6 ± 1.6 (range: 0.04–4.4) years. The background and clinical characteristics at the SPECT examinations are shown in Table 1. No significant difference was observed in the background variables between patients with symptomatic stroke and those without symptomatic stroke, except for the frequency of pyramidal tract signs. Regarding the MRI findings, the number of old lacunes was significantly higher in those with stroke than in those without stroke (*p* = 0.007). White matter hyperintensities in temporal tip and external capsule were identified in all the patients. The frequency of grade D and % volume of WMLs was higher in those with stroke than in those without stroke; however, the differences were not significant (Table 1).

**Table 1:** Comparison of the baseline characteristics and MRI findings between patients with and without stroke.

**Variable**	**All (*n =* 14)**	**With stroke (*n =* 8)**	**Without stroke (*n =* 6)**	With stroke vs. without stroke *p*-value^*^
Age (y) assessed, mean ± SD, range	56.3 ± 10.2, 41–75	57.5 ± 11.6, 41–75	54.8 ± 8.8, 46–70	0.80
Sex (male/female) *n*, (%)	9 (64.3)/5 (35.7)	6 (75.0)/2 (25.0)	3 (50.0)/3 (50.0)	0.58
Vascular risk factor *n* of combined, (%)	6 (42.9)	5 (62.5)	1 (16.7)	0.14
Hypertension *n*, (%)	1 (7.1)	1 (12.5)	0 (0.0)	1.00
Dyslipidemia *n*, (%)	4 (28.6)	3 (37.5)	1 (16.7)	0.58
Diabetes mellitus *n*, (%)	0 (0.0)	0 (0.0)	0 (0.0)	
Active smoking *n*, (%)	1 (7.1)	1 (12.5)	0 (0.0)	1.00
**Clinical manifestations**				
Number of previous stroke events (*n*) Mean ± SD, range	1.1 ± 1.4, 0-5	2.0 ± 1.3, 1–5	0	nd
Migraine *n*, (%)	4 (28.6)	1 (12.5)	3 (50.0)	0.24
Mood disorder *n*, (%)	3 (21.4)	1 (12.5)	2 (33.3)	0.54
Pyramidal sign *n*, (%)	6 (42.9)	6 (75.0)	0 (0.0)	0.001^†^
Pseudobulbar palsy *n*, (%)	4 (28.6)	4 (50.0)	0 (0.0)	0.08
Dementia (MMSE ≤ 23) *n*, (%)	3 (21.4)	3 (37.5)	0 (0.0)	0.21
**Medication**				
Lomerizine hydrochloride *n*, (%)	10 (71.4)	5 (62.5)	5 (83.3)	0.58
Antiplatelet drugs n of combined (%)	13 (92.9)	8 (100)	5 (83.3)	0.43
Cilostazol *n*, (%)	7 (50.0)	6 (75.0)	1 (16.7)	0.10
Aspirin *n*, (%)	5 (35.7)	3 (37.5)	2 (33.3)	1.00
Clopidogrel *n*, (%)	3 (21.4)	1 (12.5)	2 (33.3)	0.54
Nicergoline *n*, (%)	1 (7.1)	1 (12.5)	0 (0.0)	1.00
**MRI findings** ^‡^				
Number of lacunes (*n*), mean ± SD, range	9.7 ± 9.5, 0–30	15.4 ± 8.8, 2–30	2.2 ± 1.8, 0–5	0.007^†^
White matter lesion grade: n of A:B:C:D (% of grade D)	0:0:5:9 (64.3)	0:0:1:7 (87.5)	0:0:4:2 (33.3)	0.09
WHL/Brain volume (%), mean ± SD, range	14.2 ± 6.9, 5.3-26.4	16.0 ± 6.3, 9.1-26.4	11.7 ± 7.5, 5.3-25.2	0.16

^*^Fisher exact test for categorical data, and Wilcoxon rank sum test for numerical data (age, number of lacunes, WHL%).^†^Significant. Nd, not done.^‡^All patients showed white matter hyperintensity in the temporal tip and external capsule. White matter lesion grade; A systematic method of assessing white matter lesions reported by Chabriat et al. (A, no lesions; B, punctate or mild periventricular hyperintensities or both; C, nodular or moderate periventricular hyperintensities or both; D, confluent foci or severe periventricular hyperintensities or both) (15).

### Comparison of cerebral blood flow parameters between patients with and without stroke

**Figure 1:**
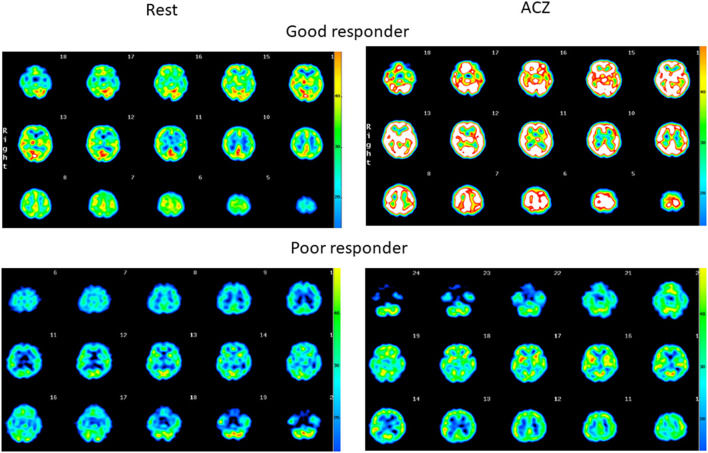
Quantitative SPECT images at rest and after ACZ injection. **(Upper)** Diffuse increase in the rCBF, indicating the maintenance in cerebrovasoreactivity in the patient (64-year-old female without stroke). **(Lower)** Only a slight increase in the rCBF, indicating little residual cerebrovasoreactivity in the patient (42-year-old male with stroke).

SPECT images of representative patients, a good responder to ACZ without symptomatic stroke (Figure 1, upper panel), and a poor responder to ACZ with symptomatic stroke (Figure 1, lower panel) are shown. Among the 16 CBF parameters, a significant difference was observed between those with and without stroke in the thalamus. ACZ-rCBF in the thalamus was significantly lower in those with stroke, at 35.6 ± 9.4, than without stroke, at 51.1 ± 7.6 (*p* = 0.007). ΔrCBF in the thalamus was significantly lower in those with stroke, at 10.6 ± 3.7, than in those without stroke, at 21.0 ± 7.9 (*p* = 0.02), but the rCVR (%) was not significantly different between the two groups (Table 2).

**Table 2:** Comparison of the cerebral blood flow parameters between patients with and without stroke.

Variable^*^	**All (*n =* 14)**	**With stroke (*n =* 8)**	**Without stroke (*n =* 6)**	With stroke vs. without stroke p-value^**^
**REST-rCBF (mL/100 g/min)**				
Cortex	26.0 ± 5.9, 12.8–37.4	26.4 ± 5.2, 20.0–37.4	25.6 ± 7.3, 12.8–34.4	0.90
Lenticular nucleus	29.6 ± 7.6, 12.6–42.4	31.3 ± 5.7, 22.2–42.4	27.2 ± 9.6, 12.6–40.6	0.30
Thalamus	27.2 ± 8.3, 13.2–38.9	25.0 ± 7.1, 15.1–37.1	30.2 ± 9.4, 13.2–38.9	0.20
Cerebellum	32.8 ± 7.1, 17.5–47.9	33.4 ± 6.6, 27.1–47.9	32.0 ± 8.4, 17.5–42.1	0.80
**ACZ-rCBF (mL/100 g/min)**				
Cortex	43.8 ± 8.4, 28.4–58.4	40.5 ± 7.8, 28.4–48.8	48.1 ± 7.6, 40.4–58.4	0.16
Lenticular nucleus	51.2 ± 9.3, 34.8–67.3	49.1 ± 8.4, 34.8 −59.0	54.0 ± 10.5, 44.9–67.3	0.52
Thalamus	42.2 ± 11.5, 23.0–63.4	35.6 ± 9.4, 23.0–53.1	51.1 ± 7.6, 44.0–63.4	0.007^†^
Cerebellum	55.1 ± 8.5, 37.1–68.4	53.6 ± 9.5, 37.1–63.5	57.0 ± 7.2, 46.0–68.4	0.90
Δ**rCBF (mL/100 g/min)**				
Cortex	17.7 ± 8.3, 6.8–32.7	14.1 ± 6.5, 6.8–23.4	22.5 ± 8.4, 11.1–32.7	0.05
Lenticular nucleus	21.6 ± 9.3, 11.2–39.6	17.8 ± 6.6, 11.2–26.9	26.7 ± 10.6, 14.3–39.6	0.07
Thalamus	15.0± 7.7, 6.1–30.5	10.6 ± 3.7, 6.1–16.1	21.0 ± 7.9, 6.8–30.5	0.02^†^
Cerebellum	22.3 ± 9.4, 5.8–39.7	20.2 ± 9.7, 5.8–32.4	25.0 ± 8.9, 15.1–39.7	0.52
**rCVR (%)**				
Cortex	76.3 ± 58.1, 18.2–254.7	55.2 ± 25.9, 18.2–92.4	104.5 ± 78.5, 37.8–254.7	0.12
Lenticular nucleus	85.9 ± 72.3, 26.4–313.6	58.3 ± 21.9, 26.4 −88.0	122.7 ± 100.4, 46.4–313.6	0.16
Thalamus	62.3 ± 51.8, 18.2–230.9	43.7 ± 13.4, 19.5–60.2	87.1 ± 73.7, 18.2–230.9	0.05
Cerebellum	75.6 ± 51.7, 18.6–226.4	63.8 ± 35.2, 18.6–119.4	91.3 ± 68.6, 45.3–226.4	0.61

^*^Mean ± SD, range. ^**^Wilcoxon rank sum test.^†^Significant. REST-rCBF, regional cerebral blood flow at rest; ACZ-rCBF, regional cerebral blood flow after acetazolamide; ΔrCBF, increase in rCBF; rCVR, regional cerebral vasoreactivity.

### Cerebrovasoreactivity in the thalamus and prognosis of patients with stroke

As described above, we found that ACZ-rCBF in the thalamus was significantly associated with past incidents of symptomatic stroke in patients with CADASIL. We further examined the relationship between ACZ-rCBF in the thalamus and the prognosis of patients with stroke. We performed a logistic regression analysis of ACZ-rCBF in the thalamus and MRI findings in association with critical events (stroke or death) during the 2 years following SPECT examination. Receiver operating characteristic analysis showed significance for ACZ-rCBF (odds ratio = 0.44, 95% CI = 0.02–0.92), but not for the number of lacunes or WHL/brain volume (Table 3).

**Table 3:** Univariate logistic regression analysis of MRI/SPECT findings in association with the recurrence of stroke or critical event during 2 years.

**MRI/SPECT findings**	**Odds ratio (95% CI)**	**ROC analysis, AUC**
Number of lacunes	1.21 (0.98, 1.88)	0.81
WHL/Brain volume (%)	1.02 (0.79, 1.32)	0.56
ACZ-rCBF (thalamus) (mL/100 g/min)	0.44 (0.02, 0.92)^*^	0.94

Eight patients with stroke were analyzed. During the 2 years after SPECT examination, four patients were free of stroke, whereas the other four suffered from stroke (n = 3) or died suddenly (n = 1). ^*^Significant.

## Discussion

In this study, we found that cerebrovasoreactivity in the thalamus was significantly lower in CADASIL patients with symptomatic stroke than in those without symptomatic stroke. A similar association was not observed in regions other than the thalamus.

Some imaging studies in patients with CADASIL have shown significant findings in the thalamus. Two diffusion tensor imaging (DTI) studies have identified significant microstructural changes in the thalamus and putamen of patients with CADASIL compared to controls (16, 17). One DTI study showed a negative correlation with the mini-mental state examination score in patients (16), and the other showed a correlation with executive function tests in patients without dementia (17). An ^18^F-FDG PET study reported that the regional cerebral metabolic rate of glucose (rCMRglc) was lower in the thalamus and caudate than in the putamen and cortical lobes in patients with CADASIL (18).

The significant cerebrovasoreactivity parameters in the thalamus were the ACZ-rCBF and ΔrCBF. Rest-rCBF and rCVR in the thalamus were not significantly different. This suggests that the maximal dilatation capacity, rather than basal flow, of cerebral small arteries may influence the occurrence of stroke.

As for location of previous symptomatic stroke, only one out of 16 cumulative prior strokes occurred in the thalamus (Supplementary Table 1), suggesting that the reduced vasoreactivity of the thalamus in eight patients with symptomatic stroke was not due to the site of previous stroke in the thalamus.

Regarding location of lacunes and microbleeds between the two groups, the number of lacunes and also microbleeds in thalamus and that in lenticular nucleus were larger in patients with stroke than in those without stroke (Supplementary Table 2). On the other hand, vasoreactivity in thalamus was decreased in patients with stroke compared with those without stroke, whereas vasoreactivity in lenticular nucleus was similar between with and without stroke (Table 2). Taken together, we think that the decreased vasoreactivity in the thalamus may reflect the disease process of small vessel in the CADASIL, and not be due solely to increased lacunes and microbleeds in the thalamus.

A previous report showed total cerebrovasoreactivity at baseline and an increase in WMH at the seven-year follow-up (10), suggesting that decreased cerebrovasoreactivity may be a potential predictor of the clinical progression of CADASIL. Based on this hypothesis, we further analyzed the recurrence of symptomatic stroke during the 2 years following SPECT in relation to cerebrovasoreactivity in the thalamus. Logistic regression analysis revealed that critical events (stroke or death) during the 2 years following SPECT examination were significantly related to ACZ-rCBF in the thalamus but not to WMH volume or the number of lacunar infarctions (Table 3). Our results suggest that ACZ-rCBF in the thalamus may be a new biomarker of CADASIL progression.

Diameter of perforating arteries supplying central gray matter lesions are similar, but the length of the thalamoperforating artery is shorter than the lenticulostriate artery or anterior choroidal artery from the anatomical construction. Anatomical construction of cerebral vessel also suggested that the thalamoperforating artery may be more easily influenced by changes of blood pressure directly more than the lenticulostriate artery or anterior choroidal artery. These factors might contribute that the thalamoperforating artery is the most sensitivity artery against ACZ to evaluate the pathological process of small vessel in CADASIL.

It is uncertain whether vasoreactivity in thalamus decreases at early stage of the disease. Comparison between patients at early stage and controls may be informative, but SPECT data of controls were not available due to the radiation exposure. Previous study compared q-space imaging values between patients with preclinical CADASIL and healthy controls and detected early neuronal change in frontal lobe and central gray matter in preclinical CADASIL (19). From the previous finding, we speculate that vasoreactivity in central gray matter, which includes thalamus, might occur from early stage of the disease.

A limitation of this study was the small number of patients, mainly because of the rare prevalence of CADASIL. In addition, we needed to identify patients who underwent ACZ-challenged SPECT using the ARG method for quantitative analysis. Another limitation was that this was a retrospective study. Further large-scale prospective studies are required to confirm our findings.

## Conclusion

In conclusion, we found that cerebrovasoreactivity in the thalamus after ACZ administration was significantly associated with stroke in patients with CADASIL. This study indicates that quantitative cerebrovasoreactivity using ACZ could be a useful marker for monitoring the disease course in patients with CADASIL.

## Data availability statement

The original contributions presented in the study are included in the article/Supplementary material, further inquiries can be directed to the corresponding author.

## Ethics statement

The studies involving human participants were reviewed and approved by the Ethics Committee of Kyoto Prefectural University of Medicine. The patients/participants provided their written informed consent to participate in this study.

## Author contributions

TM, MM, SM, and KY conceived and designed the study. MM, AH, AW-H, TK, JM, SM, HM, and TM acquired the data. MM, AH, IM, IY, AW-H, TK, JM, SM, ST, KY, and TM analyzed and interpreted the data. MM, IM, TM, and TO drafted the manuscript. All authors contributed to the article and approved the submitted version.
