# Supplementary material for: Association between cerebrovasoreactivity and stroke in cerebral autosomal dominant arteriopathy with subcortical infarcts and leukoencephalopathy
Source: Front Neurol. 2023 Jan 9;13:1087220. doi: 10.3389/fneur.2022.1087220 (PMC9868304; doi:10.3389/fneur.2022.1087220)
Supplement: Supplementary file 1 [file Data_Sheet_1.PDF]

Supplemental Table 1 The location of prior stroke in the eight patients with symptomatic stroke

Variable	
Cumulative number of prior symptomatic stroke*	
Cortex	0
Lenticular nucleus	1
Thalamus	1
Cerebellum	0
Others (Subcortical white matter, brain stem)	14
Total	16

*Strokes occurred simultaneously in multiple locations were counted separately.

Supplemental Table 2 The location of lacunes or microbleeds between patients with and without symptomatic stroke

Variable	With symptomatic stroke (n=8)	Without symptomatic stroke (n=6)
Number of lacunes		
Cortex	0	0
Lenticular nucleus	22	2
Thalamus	13	1
Cerebellum	0	0
Others (Subcortical white matter, Caudate nucleus, brain stem)	88	10
Total	123	13
Number of microbleeds *		
Cortex	0	0
Lenticular nucleus	5	0
Thalamus	14	12
Cerebellum	0	0
Others (Subcortical white matter, Caudate nucleus)	9	1
Total	28	13

* One of the eight patients with symptomatic stroke and one of the six patients without symptomatic stroke were omitted from the evaluation of for the number of microbleeds, because T2*-weighted gradient-echo planar images was not available.
